# Novel immunotargets in multiple myeloma: biological relevance and therapeutic potential

**DOI:** 10.1186/s40364-025-00799-7

**Published:** 2025-07-01

**Authors:** Jana Kotulová, Klára Baďurová, Zuzana Chyra, Sabina Ševčíková, Nikola Garbová, Tomáš Jelínek, Roman Hájek, Matouš Hrdinka

**Affiliations:** 1 Department of Haematooncology, University Hospital Ostrava, Ostrava, Czech Republic; 2 Department of Haematooncology, Faculty of Medicine, University of Ostrava, Ostrava, Czech Republic; 3 Babak Myeloma Group, Department of Pathophysiology, Faculty of Medicine, Masaryk University, Brno, Czech Republic

**Keywords:** Multiple myeloma, Aberrant plasma cells, Hematooncology, Immunotarget, Immunotherapy, Surface proteome, Surfaceomics, Biomarker discovery

## Abstract

Multiple myeloma is a hematologic malignancy characterized by complex genetic and microenvironmental factors that drive disease progression and resistance to treatment. Despite advancements in therapies targeting established antigens, such as BCMA, CD38, SLAMF7, and GPRC5D, specific challenges persist, including antigen escape, treatment resistance, and off-tumor toxicity, highlighting the urgent need for novel therapeutic modalities. Recent advances in surface proteomics and integrative omics technologies have enabled the discovery of new surface antigens with the potential to address the challenges. By targeting antigens with higher tumor specificity and lower expression in healthy tissues, emerging immunotargets offer new avenues to minimize off-tumor toxicity and reduce the risk of relapse due to antigen loss or immune evasion. This review provides an overview of emerging immunotargets, summarizing their biological functions, roles in disease pathogenesis and immune evasion, and potential for therapeutic interventions. We focused on fifteen emerging targets currently in early clinical development or the preclinical phase, highlighting LILRB4, SEMA4A, ITGB7, CCR1, and CD70 as the most promising. These immunotargets demonstrate significant potential for next-generation immunotherapies, including antibody–drug conjugates, bispecific antibodies, and chimeric antigen receptor (CAR) T-cell therapies. Preclinical or early clinical studies show favorable safety profiles, high tumor specificity, and mechanisms to overcome immune resistance, collectively suggesting the potential for improved patient outcomes and reduced adverse effects. By presenting a comprehensive summary of these advances, this review underscores the translational potential of emerging immunotargets and provides insights to guide the development of innovative therapeutic approaches to improve outcomes for multiple myeloma patients.

**Supplementary Information:**

The online version contains supplementary material available at 10.1186/s40364-025-00799-7.

## Background

Multiple myeloma (MM) is a hematologic malignancy characterized by the clonal proliferation of aberrant plasma cells in the bone marrow, leading to clinical complications such as anemia, renal failure, and bone lesions. Despite substantial advancements in therapeutic options, including proteasome inhibitors and immunomodulatory drugs, MM remains incurable, with most patients eventually experiencing relapse and resistance [1, 2]. These challenges, coupled with the rising global incidence and mortality of MM, underscore the urgent need for innovative and durable therapeutic strategies.

The rise of immunotherapy has revolutionized the MM treatment landscape. U.S. Food and Drug Administration (FDA)-approved immunotherapeutic modalities include monoclonal antibodies (mAbs), bispecific antibodies (bsAbs), antibody–drug conjugates (ADCs), and chimeric antigen receptor (CAR) T-cell therapies, as outlined in Table 1. These therapies leverage diverse mechanisms, including target blocking or inhibition with mAbs, cytotoxic payload delivery through ADCs, direct cytotoxicity mediated by CAR T cells, and immune cell recruitment and engagement through mAbs and bsAbs [3, 4]. Additionally, emerging approaches, such as natural killer (NK) cell-based therapies, further expand the immunotherapeutic toolkit [5].

**Table 1:** FDA-approved immunotherapy drugs targeting MM surface antigens (adopted from https://www.myeloma.org/multiple-myeloma-drugs, updated on February 5, 2025)

Target	Biological Function	Modality	Product Name	Company	Year
BCMA	B-cell development, binds B-cell-activating factor (BAFF)	Ab-drug conjugates	Belantamab mafodotin-blmf	GlaxoSmith-Kline	2020
		CAR T cells	Idecabtagene vicleucel	Bristol Myers Squibb	2021
			Ciltacabtagene autoleucel	Janssen	2022
BCMA/CD3	B-cell development, binds BAFF, CD3 is a TCR complex component and T-cell marker	Bispecific Ab (BCMA-directed CD3 T-cell engager)	Teclistamab-cqyv	Janssen	2022
			Elranatamab-bcmm	Pfizer	2023
CD38	Transmembrane glycoprotein, cell adhesion, enzymatic activity	Monoclonal Ab	Daratumumab	Janssen	2015
			Daratumumab and hyaluronidase-fihj	Janssen	2020
			Isatuximab-ifrc	Sanofi	2021
GPRC5D/CD3	Orphan G-protein coupled receptor, CD3 is a TCR complex component and T-cell marker	Bispecific Ab (GPRC5D-directed CD3 T-cell engager)	Talquetamab	Janssen	2023
SLAMF7	Surface receptor, immune cell activation, and adhesion via homotypic interactions	Monoclonal Ab	Elotuzumab	Bristol Myers Squibb	2015

Approved immunotherapies for MM target key surface antigens include B-cell maturation antigen (BCMA, gene ID: TNFRSF17), cluster of differentiation 38 (CD38), signaling lymphocytic activation molecule family member 7 (SLAMF7), and G protein-coupled receptor class C group 5 member D (GPRC5D). These targets have demonstrated significant efficacy across both newly diagnosed and relapsed/refractory (R/R) MM settings and continue to be the subject of extensive clinical optimization, particularly for the development of novel therapeutic modalities such as next-generation ADCs, bsAbs, and cell-based therapies (reviewed in [4, 6–8]).

Despite substantial improvements in treatment outcomes of MM, the current therapeutic strategies exploiting approved antigens are not without their limitations; challenges such as antigen escape, restricted tissue distribution, and on-target, off-tumor toxicities underscore the need for novel immunotargets with enhanced specificity, safety, and durability of response [9, 10]. To address the limitations of existing targets, significant progress has been made in leveraging surface proteomics and integrative omics-based approaches for novel target discovery. Surface proteomics utilizes mass spectrometry techniques to identify differentially expressed surface proteins on MM cells, providing a high-resolution view of the surface proteome. Integrative omics approaches, combining transcriptomics, proteomics, and bioinformatics, create a comprehensive framework for mapping surface antigen expression patterns, thereby facilitating the identification of potential immunotargets with improved specificity and therapeutic potential.

Recent foundational studies by Anderson et al. [11], Ferguson et al. [12], Yao et al. [13], and Di Meo et al. [14] have independently developed multi-omics target discovery pipelines and collectively identified dozens of MM-associated surface antigens with potential therapeutic relevance. These investigations have significantly expanded our understanding of the MM surface proteome and provide a robust foundation for the systematic evaluations of emerging immunotargets.

To date, however, no integrative overview has compared the individual hits identified in the four surfaceomics-driven studies [11–14] and evaluated their relevance across the MM immunotarget landscape. This review addresses the gap by providing a comprehensive analysis and rational ranking of identified immunotargets.

We focus on fifteen emerging targets and explore their biological roles in MM pathogenesis and immune evasion, assess their potential to overcome current therapeutic limitations, and address their current status in early translational research. Finally, we outline key directions for future studies to validate and optimize these targets, with the overarching goal of advancing MM immunotherapy and improving patient outcomes.

## Emerging immunotargets in MM

**Fig. 1:**
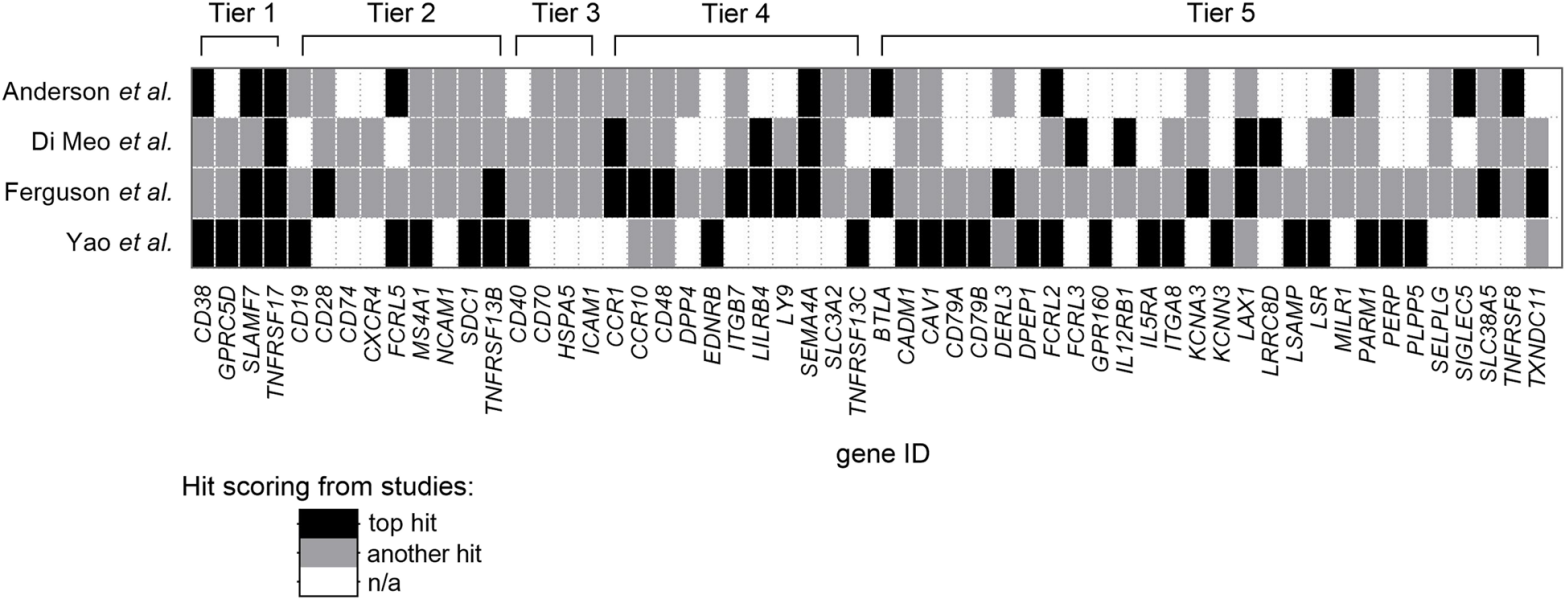
Multiple myeloma immunotargets identified in recent surfaceomics studies. This schematic categorizes the most frequently identified hits from four recent large-scale surface proteomic studies [11–14] by novelty tier and scoring. Novelty tiers are defined as follows: 1, Clinically approved immunotargets; 2, Later-stage clinical trial immunotargets (Phase II-III); 3, Early clinical development immunotargets (Phase I); 4, Preclinical development immunotargets; and 5, Unvalidated screening candidates. Scoring hit categories include: Top hit (black rectangle; antigen identified as a top target in the corresponding study); Another hit (grey rectangle; antigen identified in the screening study as a specific hit, but not designated as a top target); n/a (white rectangle; not applicable, scoring status unavailable or antigen not listed)

Based on the integration of four recent MM surfaceomics studies [11–14], we categorized the identified immunotargets into five novelty tiers reflecting their level of validation and stage of clinical development (Fig. 1, Supplementary Table S1). Tier 1 includes FDA-approved immunotargets, while Tier 2 comprises those currently undergoing advanced clinical trials. Tiers 3 and 4 represent emerging targets in early clinical and preclinical development, respectively. Tier 5 encompasses unvalidated screening candidates with no supporting preclinical data.

In this review, we focus exclusively on Tier 3 and 4 targets due to their novelty and translational potential. Tiers 1 and 2 were not relevant to the scope of this study because their clinical relevance is already being addressed in extensive ongoing trials. Likewise, Tier 5 targets were omitted from the analysis due to the lack of supporting validation data. This tiered framework allows for a focused and timely evaluation of emerging immunotargets most likely to shape the future MM treatment landscape. Although several Tier 2 targets are approaching clinical implementation, their therapeutic utility is well-characterized. In contrast, Tier 3 and 4 targets represent a high-priority frontier for research, with the potential to complement or enhance current therapies and to address persistent challenges such as antigen escape, treatment resistance, and suboptimal safety profiles.

### Cluster of differentiation 40 (CD40)

#### Biological functions

A member of the tumor necrosis factor (TNF) receptor superfamily (TNFRSF), CD40, also called TNFRSF5, is expressed on various immune and non-immune cells. TNFRSF receptors are characterized by their ability to bind TNFs through an extracellular cysteine-rich domain (CRD) [15]. CD40 is pivotal in immune responses, particularly in B-cell and dendritic cell (DC) activation [16]. The interaction between CD40 and its ligand, CD40L, which is predominantly expressed on activated CD4^+^ T cells, is essential for germinal center formation and class-switched antibody production. This interaction also triggers several signaling pathways, including NF-κB, PI3K/AKT, MAPKs, and JAK3/STATs, which drive diverse biological processes, such as immune activation, thrombosis, hematopoiesis, and tissue inflammation [17–19].

The CD40/CD40L immune checkpoint initiates bidirectional signaling, enabling activation of both innate and adaptive immune cells. CD40 activation empowers DCs to stimulate anti-tumor T cells and can reprogram macrophages to eradicate tumor stroma [17, 18]. CD40 exists in both membrane and soluble forms, with the latter produced through proteolytic cleavage or alternative splicing [19]. The shedding of CD40 from a cell surface may serve as a negative feedback mechanism, regulating its activity by reducing the levels of membrane-anchored CD40 [20].

In MM, CD40 has been detected in most tested MM cell lines and patient specimens [19]. In this context, its activation modulates MM cell proliferation and clonogenicity. Additionally, CD40 promotes MM progression by enhancing cell adhesion and migration, supporting survival within the bone marrow microenvironment [19], indicating strong involvement in MM pathogenesis.

#### Therapeutic potential and challenges

The CD40/CD40L axis is an attractive therapeutic target in various diseases, and several agents have been developed for this purpose, including agonistic and antagonistic mAbs, cellular vaccines, and adenoviral vectors. In MM, mAbs targeting CD40, such as SGN-40 (dacetuzumab), have demonstrated cytotoxic effects against MM cell lines and shown favorable safety profile, though clinical responses in trials were only modest (NCT00079716, NCT00664898) [21]. Combination therapies using CD40-targeting agents along with drugs such as lenalidomide and dexamethasone have also shown promise in R/R MM (NCT00525447) [22]. Another anti-CD40 mAb, lucatumumab, displayed anti-MM activity in preclinical tests, but limited clinical efficacy in R/R MM patients [23]. A third mAb with increased affinity, XmAbCD40, exhibited enhanced cytotoxicity against MM cell lines, but clinical studies are still pending [24].

CAR T cells have been engineered to express CD40L and have been investigated in various leukemia and lymphoma models. These CD40L-expressing CAR T cells effectively stimulate antigen-presenting cells, recruit immune effectors, and mobilize endogenous T cells to recognize and attack tumors [4, 25]. Furthermore, CAR T cells engineered to secrete anti-CD40 through non-viral vectors have shown potential for enhancing CAR T-cell efficacy in an ovarian cancer xenograft model [26]. CD40 is also a promising immunotherapy target in other cancers, including melanoma, pancreatic cancer, and mesothelioma [27].

Overall, CD40 is a compelling immunotherapy target for MM, due to its role in immune regulation and direct impact on MM cells. As summarized by Tang et al., CD40-CD40L interactions can suppress immune responses by upregulating immune checkpoint molecules, creating an immunosuppressive environment that enables MM cells to evade immune surveillance [19]. However, clinical applications have been challenged by the risk of cytokine release syndrome and hepatotoxicity. Furthermore, CD40 is expressed on normal APCs and B cells, raising concerns about off-tumor activation and systemic immune-related toxicities [28]. Therapeutic development will require a nuanced understanding of the diverse functions of CD40 and its complex involvement in the MM microenvironment. Future research should focus on elucidating the mechanisms of CD40 signaling in MM and exploring synergistic combinations with existing therapies to maximize the clinical benefits.

### Cluster of differentiation 70 (CD70)

#### Biological functions

CD70, also known as TNFSF7 or CD27 ligand (CD27L), is a TNFRSF member primarily functioning as a costimulatory molecule for immune cells [29]. CD70 is predominantly expressed in lymphoid tissues, including the thymus, spleen, peripheral lymph nodes, and gut-associated lymphoid tissues, and exhibits limited expression in non-lymphoid tissue across vital organs. This transmembrane glycoprotein plays a crucial role in lymphocyte maturation and proliferation, and is primarily expressed on mature DCs and small subsets of activated B and T cells [30]. Its primary binding partner is CD27, a receptor that regulates immune cell activity and serves as a marker of memory B cells [29]. CD27–CD70 interactions promote B-cell expansion, germinal center formation, and plasma cell differentiation in the presence of cytokines [30, 31].

Under normal physiological conditions, CD70 expression is tightly regulated. However, elevated levels of CD70 have been observed in various malignancies, and its overexpression contributes to tumor progression [32]. In non-malignant cells, CD70 supports cell proliferation by promoting cell cycle entry [31, 33]. In tumor cells, CD70 engages with CD27, resulting in secretion of soluble CD27 (sCD27) and proteolytic shedding of the CD27 ectodomain. This interaction activates the NF-κB and c-Jun kinase signaling pathways, promoting malignant cell proliferation and survival [32]. High CD70 expression is associated with poor prognosis in several cancer types, further highlighting its role in tumor pathogenesis [31, 32, 34].

#### Therapeutic potential and challenges

Earlier expression analyses revealed that approximately 60% of tested MM cell lines exhibited CD70 expression, suggesting it as a viable target for therapies against B-lineage malignancies [30]. Forster et al. demonstrated that the interaction between CD70 and CD27 promotes plasma cell survival, leading to enhanced MM cell proliferation. Notably, the blockade or knock-out of CD70 resulted in significant inhibition or complete cessation of growth in CD70-expressing MM cells [35]. A recent study of Wang et al. evaluating CD27 expression profile in MM patient samples, confirms the previous findings. This study demonstrated poorer overall survival correlated with CD27 levels and identified that MM cells rely on the CD27–CD70 axis to evade immune surveillance, hence promoting cancer progression [36]. Notably, CD70 is also significantly upregulated in extramedullary MM [37].

Currently, CD70-targeted therapy development is mainly focused on mAbs, both as single agents and in combination with other treatments. The most advanced anti-CD70 mAb, cusatuzumab (ARGX-110), is designed to inhibit CD70/CD27 signaling. Cusatuzumab exerts antitumor effects through enhanced antibody-dependent cellular cytotoxicity, complement-dependent cytotoxicity, and antibody-dependent cellular phagocytosis [32, 38, 39]. Notably, a mAb with a significantly higher affinity for CD70, IMM40H, has shown greater therapeutic efficacy in preclinical studies compared to other anti-CD70 antibodies, including cusatuzumab. IMM40H exhibited potent Fc-dependent effector functions and significant antitumor activity in the U266B1 myeloma xenograft model, eradicating tumors at low doses [32]. Another anti-CD70 antibody, SGN-70, has shown potent anti-MM effects in vitro, and significantly prolongs the survival of tumor-bearing mice by lysing CD70-expressing malignant cells through Fc-dependent mechanisms [40]. In addition to mAbs, a current clinical study (NCT04662294) is evaluating the use of anti-CD70 CAR T cells to treat hematologic malignancies. These CAR T cells have demonstrated potential in targeting CD70-positive malignant cells, providing a promising approach for treating cancers with high CD70 expression.

CD70 is in general an attractive immunotherapeutic target due to its limited expression in normal tissues and frequent upregulation in malignancies; however, its clinical application is strongly challenged by potential off-target effects and CD27 co-expression in hematologic cancers. Importantly, CD70 is transiently expressed on activated T cells, leading to fratricide during the generation of CD70-targeting CAR T cells. This results in impaired T cell expansion and function. Additionally, off-tumor expression of CD70 on activated immune cells raises concerns about immune-mediated toxicities. Strategies such as CD70 gene knockout in T cells have been employed to overcome these obstacles and enhance the efficacy and safety of CD70-directed therapies [41]. Further research is required to optimize these therapies and explore combination strategies to enhance therapeutic efficacy and safety.

### Heat shock protein family A (Hsp70) member 5 (HSPA5)

#### Biological functions

Heat shock protein family A (Hsp70) member 5 (HSPA5), also known as glucose-regulated protein 78 (GRP78) or binding immunoglobulin protein, is a key member of the HSP70 family. In normal cells, HSPA5 is primarily localized to the endoplasmic reticulum (ER) membrane, where it plays a crucial role in protein folding, assembly, and degradation, serving as a significant chaperone regulating the unfolded protein response [42, 43].

Additionally, HSPA5 contributes to tumor cell survival and chemoresistance by facilitating the correction of misfolded proteins and enabling recovery from ER stress, making it an attractive target for therapeutic inhibition in cancer cells [44, 45]. Although it predominantly resides in the ER, HSPA5 can translocate to the tumor cell surface under stress conditions, a phenomenon rarely observed in normal cells [46, 47]. Once on the cell surface, HSPA5 interacts with extracellular ligands, such as alpha-2-macroglobulin (α2M) and prostate apoptosis response 4 (PAR-4), activating multiple signaling pathways involved in malignant proliferation, survival, angiogenesis, and metastasis, including ERK1/2, PI3K/Akt, and Wnt/β-catenin [48].

In MM, HSPA5 is highly expressed and plays critical roles in malignant cell adhesion and invasion, and is overexpressed in quiescent myeloma cells that are often resistant to treatment [49]. Moreover, surface-expressed HSPA5 can be released into the extracellular space when the ER calcium pool is disrupted, triggered by stress or saturation of KDEL receptors [50].

#### Therapeutic potential and challenges

HSPA5 has been detected on the surface of various malignant cells, including in prostate cancer, ovarian cancer, lymphoma, neuroblastoma, and lymphoblastic leukemia [48]. In MM, HSPA5 is overexpressed in patient samples, and its expression increases with disease progression, highlighting its potential as an immunotherapy target [51, 52].

Several HSPA5-targeting therapeutic modalities are under investigation, including the human IgM antibody PAT-SM6, which has shown promising results in combination therapies for R/R MM, both in vitro and in vivo [51, 52]. A Phase I study (NCT01727778) demonstrated the safety and tolerability of PAT-SM6 in R/R MM patients [53]. However, while the study confirmed its safety profile, it did not establish significant therapeutic efficacy, suggesting that PAT-SM6 may be more effective in combination therapy than as a single agent [53]. Additionally, HSPA5 has been implicated in resistance to the proteasome inhibitor bortezomib, further emphasizing its role in MM pathogenesis and treatment resistance [54]. In a recent study, the specific HSPA5 inhibitor HA15 was found to inhibit MM cell line growth, and displayed a synergistic effect when combined with bortezomib, significantly reducing MM cell viability by inducing ER stress [42].

When designing therapeutic approaches, the existence of intracellular, cell surface, and soluble forms of HSPA5 must be considered. For example, targeting cell surface expression of HSPA5 on acute myeloid leukemia (AML) blasts with CAR-T cells has shown promising anti-leukemic activity without affecting hematopoietic progenitor cells, indicating a potential therapeutic window. However, the authors observed antigen-dependent T cell differentiation and fratricide during CAR T cell manufacturing. The use of dasatinib during CAR T cell production has been effective in mitigating this issue, enhancing the safety and efficacy of the therapy [55]. In MM, HSPA5 is overexpressed in quiescent MM cells, making it a potential target for eliminating treatment-resistant cells [51]. Thus, targeting HSPA5, whether as monotherapy or combined with existing treatments, offers a compelling strategy for overcoming treatment resistance and improving MM therapy outcomes.

### Intercellular adhesion molecule 1 (ICAM-1)

#### Biological functions

Intercellular adhesion molecule 1 (ICAM-1), also referred to as CD54, is a transmembrane glycoprotein belonging to the immunoglobulin superfamily that binds to key ligands such as LFA-1 (CD11a/CD18) and Mac-1 (CD11b/CD18) [56]. ICAM-1 can undergo proteolytic cleavage, releasing a soluble form (sICAM-1) into the extracellular environment [57]. It is pivotal in mediating cell–cell interactions, particularly in the immune system, by facilitating leukocyte adhesion and transmigration across the endothelium.

Beyond its physiological functions, ICAM-1 has garnered significant attention in oncology. ICAM-1 is overexpressed in various malignancies, including colorectal cancer, lung cancer, breast cancer, and MM. In solid tumors, ICAM-1 facilitates tumor cell adhesion to the endothelium, promoting invasion and metastasis [58]. In MM, it contributes to the aberrant adhesion of plasma cells to bone marrow stromal cells, promoting tumor growth and survival. This adhesion activates signaling pathways that confer resistance to chemotherapy, a phenomenon known as cell adhesion–mediated drug resistance (CAM-DR) [59].

#### Therapeutic potential and challenges

ICAM-1 serves as a critical mediator in tumor progression and metastasis across various cancer types. Its consistent overexpression in malignancies, including MM, supports its potential as a therapeutic target. However, challenges remain, including the reported occurrence of sICAM-1, which may act as a decoy receptor, limiting therapeutic efficacy. Clinical application faces challenges due to ICAM-1's expression on normal endothelial and immune cells, raising concerns about off-tumor effects. Moreover, ICAM-1 expression can be upregulated in inflammatory conditions, potentially leading to unintended targeting of non-malignant tissues. Despite these concerns, studies have shown that ICAM-1 CAR-T cells did not cause significant damage to essential tissues in animal models, suggesting a favorable safety profile [60]. Additionally, ICAM-1-specific CAR T cells have demonstrated robust antitumor activity in preclinical models, such as in triple-negative breast cancer [61].

The overexpression of ICAM-1 in MM potentially presents a viable target for therapeutic interventions. As a result, several clinical studies have explored therapies targeting ICAM-1 in MM. BI 505, a human mAb against ICAM-1, has been investigated in clinical trials. A Phase I trial focused on R/R MM (NCT01025206), while a Phase II study (NCT01838369) evaluated its potential in smoldering myeloma. Despite being well-tolerated, the Phase II study was terminated due to a lack of clinically relevant efficacy. Additionally, a Phase I/II study (NCT02756728) examined BI 505 in combination with high dose melphalan and autologous stem cell transplantation. Importantly, this study was terminated by the FDA following a cardiopulmonary adverse event. VP301, a bispecific CD38 and ICAM-1 antibody, was designed to leverage the overexpression of these antigens on MM cells to enhance immune-mediated tumor destruction. A Phase I trial (NCT05698888) aimed to assess the safety, tolerability, and pharmacokinetics of VP301 in patients with R/R MM. However, the study was terminated after enrolling only two participants, with no specific reason provided. To date, cell-based therapies targeting ICAM-1 have not been developed for MM. All in all, clinical trials have often reported either a lack of efficacy or adverse effects associated with ICAM-1-based modalities, advocating for developing more effective therapies.

A further obstacle is represented by the ability of cancer cells to downregulate ICAM-1 expression as a strategy for immune evasion, thus diminishing the immune-mediated cytotoxicity [62]. Interestingly, certain epigenetic therapies, such as decitabine, have been shown to upregulate ICAM-1 expression on tumor cells, enhancing their susceptibility to immune cell-mediated lysis [62]. Ren et al. also demonstrated that tumors might exploit resistance mechanisms to ICAM-1-mediated immune responses, further contributing to immune escape [63].

### C–C motif chemokine receptor 1 (CCR1)

#### Biological functions

C–C motif chemokine receptor 1 (CCR1), also known as CD191, is a highly promiscuous G protein-coupled receptor (GPCR) that is activated by at least nine chemokines, including C–C Motif Chemokine Ligand 3 (CCL3) (MIP-1α), CCL5 (RANTES), CCL7 (MCP-3), and CCL23 (MPIF-1). As a GPCR, CCR1 modulates several signaling pathways, including those involving adenylyl cyclase (AC), phospholipase C (PLC), and β-arrestin signaling [64–66]. CCR1 is primarily expressed in immune cells and plays a critical role in recruiting these cells to sites of inflammation, facilitating monocyte and lymphocyte migration [67].

In recent surface proteomics studies, CCR1 has emerged as one of the top six MM surface antigens, exhibiting high expression in samples from R/R MM patients and undetectable-to-minimal expression in normal tissues [14]. While CCR1 has not consistently ranked as a top hit in other studies, it has scored high as a potential MM target due to its differential expression patterns [11, 12]. Notably, activated T cells and hematopoietic stem cells show low CCR1 expression, at levels even lower than those observed for established MM targets, like BCMA, suggesting its potential to selectively target MM cells while sparing healthy tissues. Additionally, the regulatory mechanisms of CCR1 and its ligands in immune cells are believed to function similarly in MM cells, indicating that CCR1 contributes to physiological and pathological processes within the tumor microenvironment (TME) [68, 69].

#### Therapeutic potential and challenges

Emerging evidence identifies CCR1 as a promising therapeutic target in MM. Zeissig et al. recognized CCR1 expression as an independent prognostic marker in newly diagnosed MM patients and a key regulator of MM plasma cell dissemination from bone marrow in murine xenografts [70]. Furthermore, CCR1 expression is upregulated in MM cells under hypoxic conditions through hypoxia-inducible factor-2α (HIF-2α) regulation and is correlated with poor prognosis among newly diagnosed MM patients. Increased CCR1 expression is also associated with enhanced recirculation of MM cells, which may contribute to disease progression and metastasis [71].

A 2024 study further correlated CCR1 expression with the sensitivity of MM cell lines to bortezomib, although the underlying mechanism remains unclear [72]. Previous studies have also implicated CCR1 in aberrant plasma cell homing and MM lesion development [73]. Targeting CCR1 with specific antagonists has been proposed as a strategy to inhibit MM cell migration and survival [64, 74].

Gilliland et al. demonstrated that chronic CCR1 activation depends on β-arrestin-2, a mechanism that could potentially be exploited to regulate receptor turnover and surface availability [75]. Leveraging this dynamic regulation could provide opportunities for novel cell-based therapies or developing new cell engagers targeting CCR1. Moreover, the biased signaling properties of CCR1 might be exploited to design selective therapeutics that limit off-target effects while enhancing therapeutic efficacy [66]. Future research should focus on validating these therapeutic strategies in clinical settings to evaluate their impact on MM progression and treatment resistance.

Clinical application of CCR1 faces challenges due to its expression on normal immune cells, raising concerns about off-tumor effects. Additionally, the redundancy and complexity of the chemokine network, as well as species-specific differences in CCR1 function, complicate the translation of preclinical findings to human therapies. Despite these hurdles, the favorable safety profile observed in early-phase clinical trials of CCR1 antagonists for non-oncologic indications suggests potential for their use in cancer therapy, warranting further investigation [65, 76]. Considering its dual involvement in immune cell recruitment and MM pathogenesis, CCR1 represents an ambiguous target for further immune-based therapies.

### C–C motif chemokine receptor 10 (CCR10)

#### Biological functions

C–C motif chemokine receptor 10 (CCR10), a member of the CCR family, functions as a GPCR (25, 26). Primarily associated with the PI3K/Akt/mTOR pathway [79], CCR10 signaling regulates a variety of cellular processes. CCR10 is expressed on both T and B cells, and plays pivotal roles in T-cell trafficking, immune activity, and immune responses to viral infections [80].

CCR10 has two known endogenous ligands: CCL27 and CCL28. CCL27, primarily produced by keratinocytes, enhances gene expression in actin cytoskeleton remodeling upon binding CCR10, promoting cell migration and morphological changes. CCL28 is a structurally similar chemokine produced in mucosal tissues, which plays a key role in normal mucosal immune function by promoting the migration of IgA-producing plasma blasts [77, 78]. Together, these ligands facilitate immune cell trafficking to target tissues [80].

Although CCR10 is typically expressed on immune cells, it has also been detected at elevated levels in various tumor cells, including malignant melanoma and squamous cell carcinoma, where its overexpression is linked to poor prognosis [81, 82]. Notably, CCR10 could also potentially inhibit tumor growth and metastasis by modulating processes related to invasiveness and epithelial-mesenchymal transition (EMT), making it a dual player in cancer biology [78].

#### Therapeutic potential and challenges

Recent screening studies highlight CCR10 as a potential therapeutic target in MM. Ferguson et al. demonstrated that CCR10 is widely expressed on the surface of MM cells, exhibiting significantly higher expression compared to other B-cell malignancies [12]. Moreover, elevated CCR10 expression in CD138-positive plasma cells from MM bone marrow aspirates was correlated with poorer overall survival [12]. Notably, CCR10 expression was not detected in hematopoietic cells from peripheral blood, suggesting a selective expression profile in MM cells [12].

Thangavadivel et al. further showed that an increased concentration of CCL27, a natural ligand of CCR10, is associated with bone marrow homing of malignant cells in MM [83]. High levels of CCL27 found in MM patient samples negatively correlated with overall survival and positively correlated with early resistance to bortezomib treatment. The authors demonstrated that CCL27 effects depend on CCR10 expression. In an innovative therapeutic approach, they developed a CAR T-cell product targeting CCR10 using CCL27, which exhibited in vitro cytotoxicity against the MM.1S cell line. Interestingly, the off-tumor expression of CCR10 was comparable to the expression of other MM targets currently under investigation (e.g., SLAM7 and CD138), suggesting a potentially favorable safety profile [12, 84, 85]. These findings indicate that targeting CCR10 may offer a novel therapeutic avenue for MM, with potential advantages in terms of safety and efficacy.

However, challenges remain in fully understanding the complex role of CCR10 in MM pathophysiology, including balancing its functions in normal immune cell trafficking and its potential pro-tumorigenic effects. Further investigations into this receptor’s downstream signaling pathways and interaction networks are essential for optimizing CCR10-targeted therapies and assessing their clinical potential in MM.

### Cluster of differentiation 48 (CD48)

#### Biological functions

CD48, also referred to as SLAMF2, is a glycosylphosphatidylinositol-anchored membrane protein and a member of the CD2 subset of the immunoglobulin superfamily. It is expressed on nearly all hematopoietic cells, including T cells, B cells, NK cells, DCs, and other immune cells [86, 87]. CD48 lacks a cytoplasmic domain but still engages in cell–cell interactions that influence various immune functions. Primarily serving as a ligand for CD2 and SLAMF4 (also known as 2B4), CD48 facilitates immune cell adhesion, a central process for immune activation and signaling [88]. Upon binding CD2, CD48 helps form immune synapses and supports T-cell receptor (TCR) signaling through associations with membrane microdomains, thereby promoting T-cell activation and proliferation [89]. Additionally, CD48 binds to SLAMF4 to modulate NK cell activity, with outcomes dependent on the signaling context, such as SLAMF4 expression, adaptor protein presence, and receptor crosslinking extent [87].

Beyond its role in immune cell signaling, CD48 has also been implicated in modulating the TME. Pan-cancer analyses reveal elevated CD48 expression across multiple tumor types relative to normal tissue [90]. High levels of TME-associated CD48 have been proposed as a potential target in cancer immunotherapy, as CD48 expression in the TME might contribute to immune regulation and tolerance [91]. In addition to membrane-bound CD48, soluble CD48 has been detected in blood, with increased levels observed in lymphoproliferative diseases, Epstein-Barr virus infection, and arthritis [88, 92]. This soluble form suggests CD48 may play a broader role in systemic immune regulation.

#### Therapeutic potential and challenges

CD48 has emerged as a potential immunotherapeutic target for MM, due to its high expression on MM cells and critical role in immune cell interactions. Several studies have demonstrated high and stable CD48 expression in primary MM samples and in R/R MM [93, 94]. Notably, CD48 was identified as one of the top MM immunotargets identified in a recent unbiased membrane proteomics study, underscoring its therapeutic potential [12].

In preclinical studies, ADCs and mAbs targeting CD48 have yielded promising results [94–96]. For example, SGN-CD48A, a humanized anti-CD48 mAb conjugated to monomethyl auristatin E, has demonstrated potent antitumor activity against a range of human MM cell lines in vitro, while having minimal effects on resting human B, NK, and T lymphocytes [95]. Moreover, in vivo studies in mouse xenograft models have shown that SGN-CD48A induced complete remission in multiple models, indicating its suitability as a stand-alone or combination therapy in MM [95, 96]. Additionally, CD48 expression has been shown to influence the efficacy of existing MM therapies. For instance, Liu et al. reported that CD48 expression status influenced the response to daratumumab, suggesting synergy between CD48-targeted therapies and other MM treatments [5].

Despite these promising findings, concerns have been raised about the potential hematologic toxicity of CD48-targeted therapies, due to widespread CD48 expression on normal lymphocytes and monocytes. This off-tumor expression increases the risk of cytopenia and immunosuppression, which could limit the feasibility of long-term treatment. These concerns are exemplified by the early termination of the SGN-CD48A Phase I clinical trial (NCT03379584), owing to its overall benefit-risk profile. However, the high density and selective expression of CD48 on hematopoietic cells still make it a strong candidate for an avidity-based approach, where enhanced CAR T-cell “locking on” could be leveraged to promote more effective targeting of MM cells. This approach aims to enhance CAR T-cell dwell time on tumor cells by exploiting the high abundance of CD48, thereby improving therapeutic efficacy [84].

Moreover, the roles of CD48 in promoting immune cell adhesion and activation further support its potential as a therapeutic target. In an immunomodulatory context, CD48 co-recruitment by a bispecific programmed death-1 (PD-1)/CD48 antibody was found to enhance PD-1 activation, suggesting potential synergy between CD48-targeted therapies and checkpoint inhibitors for improved antitumor effects [97]. Furthermore, CD48 contributes to immune escape by interacting with CD244 on NK cells, leading to NK cell dysfunction and reduced cytotoxicity against tumor cells [87]. Given the context-dependent regulation of CD48 in immune responses, it is essential to carefully balance its targeting to maximize therapeutic benefits while minimizing off-target effects.

### Dipeptidyl peptidase 4 (DPP4)

#### Biological functions

Dipeptidyl peptidase 4 (DPP4), or CD26, is a transmembrane glycoprotein belonging to the serine protease family. It comprises a short cytoplasmic domain, a transmembrane region, and an extracellular domain with dipeptidyl peptidase activity [98, 99]. As a proteolytic enzyme, DPP4 plays a critical role in the bone marrow microenvironment by modulating the degradation and inactivation of bioactive peptides, signaling molecules, and extracellular matrix components [100].

One well-characterized function of DPP4 is the inactivation of circulating hormones, such as incretins. However, its biological effects are heterogeneous and context-dependent, showing substantial variation across different cell types and tissues. DPP4 is widely expressed on multiple cell types, including T lymphocytes, B cells, NK cells, DCs, endothelial cells, and epithelial cells [101, 102]. Its surface expression has also been detected on tumor cells, where DPP4 can exhibit dual roles, acting either as a tumor promoter (e.g., in pleural mesothelioma) or as a tumor suppressor (e.g., in melanoma and neuroblastoma) [98, 103].

Within the context of MM, DPP4 is highly expressed in human osteoclasts within osteolytic bone lesions and in MM cell lines co-cultured with human osteoclasts [104]. The interaction between DPP4 and the bone marrow microenvironment is thought to drive MM cell proliferation and osteoclast formation, thereby contributing to disease progression. In other hematological malignancies, DPP4 expression is associated with increased cell proliferation and invasiveness, suggesting a broader role in promoting malignant phenotypes.

#### Therapeutic potential and challenges

DPP4 has garnered attention as a potential therapeutic target in MM, due to its expression profile and involvement in MM cell growth and bone disease. Preclinical studies have shown that targeting DPP4 with mAbs can inhibit tumor growth. For instance, anti-DPP4 mAbs (e.g., IF7 and 14D10) inhibited the growth of DPP4-positive tumor cells in T-cell lymphoma models [104, 105]. Similarly, a DPP4-targeting humanized mAb (huCD26mAb) exhibited cytotoxic activity against MM cells and also inhibited their adhesion to bone marrow stromal cells, a key mechanism that supports MM cell growth and survival in the bone marrow niche [104]. These findings suggest that DPP4-targeted therapies could disrupt critical interactions within the bone marrow microenvironment, providing a novel strategy to limit disease progression.

However, there are several important considerations when targeting DPP4 in MM. One major challenge is its broad expression across various cell types, possibly leading to unintended effects on healthy tissues and disruption of immune functions. In this regard, DPP4 inhibitors have been linked to a higher incidence of certain infections, including upper respiratory and urinary tract infections [102]. Another obstacle could be the presence of a soluble form of DPP4 found in various body fluids, including peripheral blood, urine, semen, and synovial fluid [101]. Soluble DPP4 is shed from multiple cell types, including human adipocytes, smooth muscle cells, and T helper 17 (Th17) lymphocytes [106, 107]. This soluble form may act as a decoy, potentially reducing the efficacy of DPP4-targeted therapies. On the other hand, circulating soluble DPP4 has been studied as a biomarker in various cancers and may prove useful for patient stratification and monitoring therapeutic responses [98, 102].

Overall, while DPP4-targeted therapies have shown promise in preclinical models, further investigation is needed to fully elucidate their impact on the immune system. Additionally, the role of soluble DPP4 on therapeutic efficacy remains to be elucidated. Efforts to optimize strategies that selectively target the membrane-bound form of DPP4 while minimizing off-target effects appear to be essential for advancing DPP4-based therapies in MM.

### Endothelin receptor type B (EDNRB)

#### Biological functions

Endothelin receptor type B (EDNRB or ETB), is a GPCR activated by its endogenous peptide ligand, endothelin. Endothelin, a vasoconstricting peptide, has three isoforms (endothelin-1, -2, and -3) that play critical roles in vascular homeostasis, primarily through their interactions with EDNRA and EDNRB receptors [108]. While ENDRA has a higher affinity for endothelin-1, EDNRB binds all three isoforms with similar affinity. Upon activation, EDNRB couples to various G-proteins, including Gα_i/o_, which inhibits AC; Gα_q/11_, which stimulates PLC; and Gα_12/13_, which modulates actin remodeling [108, 109]. The crystal structure of EDNRB complexed with its ligand provided deeper insights into its structure–function relationship [110].

EDNRB has been extensively studied in several cancers, including breast cancer [111] and melanoma [112]. Its role in MM has only recently been explored, with studies demonstrating its overexpression in MM patient samples compared to healthy donors. EDNRB expression is regulated by autocrine and paracrine stimulation from endothelin-1 produced by bone marrow niche cells, such as mesenchymal stromal cells [113]. Notably, EDNRB surface expression is low on normal bone marrow plasma cells and B lymphocytes, compared to its elevated expression on MM cells, suggesting its selective upregulation in aberrant plasma cells. Several recent proteomic screening studies have confirmed this unique expression pattern, highlighting EDNRB as a potential MM-specific target [14, 114, 115].

#### Therapeutic potential and challenges

Although the therapeutic potential of targeting EDNRB in cancer has been explored, only one clinical trial focused specifically on EDNRB inhibition (NCT02442466). This trial evaluated the EDNRB antagonist BQ-788 in melanoma but was terminated due to patient recruitment challenges. Despite this setback, the role of EDNRB in cancer biology remains promising. In MM, EDNRB activation drives epidermal growth factor receptor (EGFR) transactivation. It activates downstream Ras/Raf/MEK/ERK signaling pathways crucial for tumor cell proliferation and survival [116]. Macitentan, a dual endothelin receptor antagonist approved for pulmonary arterial hypertension treatment [117], has shown antitumor effects in MM, reducing the number of MM cells infiltrating bone marrow in mouse xenograft models [118]. This finding suggests that EDNRB inhibition might disrupt supportive interactions between MM cells and the bone marrow microenvironment.

Moreover, mAbs targeting EDNRB have been successfully developed, with potential applications in diagnostics and in vivo imaging [119]. These mAbs have been evaluated in preclinical studies for MM, showing potential utility for tracking MM cells in bone marrow [114]. Interestingly, despite promising results from EDNRB-targeting antagonists and mAbs, a study using CRISPR/Cas9 technology revealed that MM cell survival does not depend on EDNRB expression [114]. This finding indicates that, while EDNRB may contribute to MM pathogenesis, its inhibition alone may not be sufficient for effective MM control, warranting further research to define its precise role.

The high EDNRB expression in the brain poses another potential challenge for therapeutic targeting. EDNRB activation in the brain has been linked to neurogenesis and angiogenesis [120], which may complicate the systemic administration of EDNRB antagonists due to possible off-target effects. Additionally, interactions between EDNRB and other signaling pathways, such as the C-X3-C motif chemokine ligand 1 (CX3CL1)/C-X3-C motif chemokine receptor 1 (CX3CR1) axis in experimental hepatopulmonary syndrome, underscore the need for caution in therapeutic targeting [121]. Understanding these interactions to avoid adverse effects and develop safer therapeutic strategies will be crucial.

Overall, while selective EDNRB expression in MM cells compared to normal hematopoietic cells suggests its potential as a therapeutic target, the complexity of its signaling and expression profile highlights the need for further investigations. Exploring combination strategies, such as EDNRB inhibition alongside other MM-specific therapies, may yield more effective outcomes while minimizing potential side effects.

### Integrin β7 (ITGB7)

#### Biological functions

Integrin β7 (ITGB7) is a critical adhesion molecule primarily expressed on subsets of lymphocytes, including T cells, B cells, and NK cells. ITGB7 forms a heterodimer specifically with integrin α4 (ITGA4) or αE (ITGAE) subunits. As part of the heterodimer α4β7, it plays a pivotal role in the trafficking and homing of immune cells to specific tissues, particularly the gastrointestinal tract [122]. ITGB7 binds to its endothelial ligand, Mucosal vascular addressin cell adhesion molecule 1 (MAdCAM-1), facilitating lymphocyte migration into inflamed or damaged tissues. Further, vascular cell adhesion molecule-1 (VCAM-1) also interacts with α4β7, contributing to immune cell adhesion and migration under certain conditions [123]. For example, during the inflammation process, or specifically in the bone marrow microenvironment, VCAM-1 enhances interaction with α4β7 and promotes the retention and migration of immune cells. Another integrin heterodimer, αEβ7, binds to E-cadherin, promoting the retention of T cells within epithelial sites, particularly in the gut epithelium [124]. These processes are essential for maintaining immune surveillance and the mucosal immune response.

Similar processes are exploited in pathological conditions. In addition to inflammatory bowel diseases, such as Crohn’s disease and ulcerative colitis, ITGB7 plays a significant role in the pathogenesis of MM [125]. Mechanistically, ITGB7 enhances the adhesion of MM cells to bone marrow stromal cells and extracellular matrix components, like fibronectin and E-cadherin, via interaction with MAdCAM-1. This adhesion contributes to CAM-DR, creating a protective niche that shields tumor cells from immune surveillance. As a result, MM cells become less responsive to treatments like bortezomib and melphalan [124].

#### Therapeutic potential and challenges

Therapeutically, targeting ITGB7 presents a promising strategy. In MM, ITGB7 overexpression is associated with poor prognosis and has been particularly noted in high-risk MM subgroups, such as those with t(14;16) and t(14;20) translocations. Interestingly, the study by Choudhury et al. also suggests that epigenetic mechanisms play a role in the regulation of ITGB7 expression [126].

The rationale for targeting ITGB7 has been established in other diseases. TRK-170, an ITGB7 inhibitor, is currently in Phase II clinical trial (NCT01345799) for moderate to severe Crohn’s disease. At the same time, Vedolizumab, an FDA-approved mAb targeting α4β7 integrin, is an established treatment for Crohn's disease and ulcerative colitis. Both have demonstrated clinical efficacy and a favorable safety profile, offering insights for MM therapies.

In MM specifically, several promising therapies targeting ITGB7 are currently under investigation. For example, OPC-415, an investigational therapy targeting ITGB7, is currently being evaluated in a Phase I/II clinical trial (NCT04649073) for patients with R/R MM, specifically those with MMG49 antigen-positive myeloma cells. By inhibiting ITGB7, this therapy aims to disrupt the interactions contributing to tumor growth and resistance mechanisms, potentially offering a novel approach for treatment-resistant MM. Furthermore, CAR T cells engineered to recognize the active conformation of ITGB7 have demonstrated cytotoxic activity against MM cells in preclinical models, providing another potential avenue for treatment [121].

The development of ITGB7-targeted therapies, such as OPC-415 and TRK-170, underscores the versatility of ITGB7 as a therapeutic target across a wide range of diseases, including autoimmune disorders and hematologic malignancies. Moreover, the safety profiles of TRK-170 and Vedolizumab provide compelling evidence for the clinical viability of targeting ITGB7. Notably, targeting ITGB7 has been shown to disrupt tumor-protective interactions, enhance immune cell infiltration, and overcome immune evasion mechanisms [127–129]. However, its heterogenous expression across malignant cell populations suggests that ITGB7 is not a universal antigen in MM, but rather a high-risk associated immunotarget. Additionally, its physiological expression on gut-homing T and B cells raises concerns about possible on-target, off-tumor toxicity, particularly related to gastrointestinal immune dysregulation. Nevertheless, the collective findings highlight the potential of ITGB7 as a transformative therapeutic target.

### Leukocyte immunoglobulin-like receptor B4 (LILRB4)

#### Biological functions

Leukocyte immunoglobulin-like receptor B4 (LILRB4), also known as immunoglobulin-like transcript 3 (ILT3), is a member of LILRB family and is primarily involved in mediating immunotolerance. Its immunosuppressive functions are attributed to immunoreceptor tyrosine-based inhibitory motifs (ITIMs) shared by the LILRB family and other inhibitory receptors [130].

The transmembrane glycoprotein LILRB4 is predominantly expressed on myeloid lineage cells, such as macrophages, monocytes, and DCs, where it suppresses their activation [131]. In T cells, LILRB4 enhances differentiation into CD8 ^+^ T suppressor cells in vitro [132]. LILRB4 is also expressed on specific B-cell subsets, particularly memory and marginal zone B cells [133]. Under physiological conditions, LILRB4 expression is highly restricted to these cell types; however, its overexpression has been observed in various malignancies, including advanced-stage colorectal cancer [134], non-small-cell lung cancer [135], and ovarian tumors [136]. LILRB4 also exists in a soluble form detected in the serum of patients with pancreatic carcinoma, colorectal cancer, and melanoma [137]. Both membrane-bound and soluble forms suggest that LILRB4 plays a complex role in immune regulation and tumor progression.

Pathological LILRB4 expression has also been reported in hematologic malignancies associated with immune evasion and poor prognosis [13, 138, 139]. In MM, LILRB4 is overexpressed in patient samples and has been implicated in modulating the immune microenvironment [12]. A current study by Xie et al. provides additional mechanistic evidence showing that LILRB4 maintains the proliferation capacity of MM cells [140].

#### Therapeutic potential and challenges

LILRB4 has emerged as a potential immunotherapeutic target due to its immunosuppressive role and pathological expression in several malignancies. In hematologic cancers, LILRB4 has been identified as a marker for monocytic AML and implicated in tumor cell infiltration and T-cell suppression [138, 139]. Importantly, LILRB4 contributes to immune escape by recruiting myeloid-derived suppressor cells, which suppress T-cell function [141].

Consequently, various LILRB4-targeting therapeutic modalities are under development and have entered clinical trials. For example, mAb IO-202 is being tested for AML and chronic myelomonocytic leukemia (CMML) in a first-in-human Phase I trial (NCT04372433). Available data show good tolerability and at least moderate therapeutic effects [142]. A LILRB4-targeting ADC has shown the potential to selectively eliminate AML cells without affecting normal progenitor cells, further supporting a favorable safety profile [138].

LILRB4 has also been investigated as a target for cell-based therapies. Anti-LILRB4 CAR T cells have demonstrated potent anti-cancer effects in vitro and in xenograft mouse models of AML, without toxicity towards normal hematopoietic cells [143]. Additionally, a Phase I clinical trial (NCT04803929) is currently evaluating CAR T therapy for R/R AML. A novel biparatopic synthetic TCR and antigen receptor called STAR-T targeting LILRB4 completed a Phase I clinical trial for R/R AML (NCT05518357, results not yet available) [144].

In the context of MM, no modality has concluded a clinical trial. However, a similar STAR-T therapeutic is intended for R/R MM (NCT05913804), stemming from the recent study by Gong et al. [145]. Moreover, the work of Di Meo et al. provides a rationale for using LILRB4 as a prognostic marker, confirming its selective expression on MM cells compared to healthy tissues [14]. The authors further developed and validated a novel bispecific T-cell engager targeting LILRB4, which shows efficacy in vitro and in vivo, further supporting the potential use of LILRB4 as a therapeutic target in MM. LILRB4 expression is negatively correlated with the overall survival of MM patients and positively correlated with the severity of bone lesions [146].

While the preclinical and early clinical data from LILRB4-targeted therapies are encouraging, caution is warranted due to the potential adverse effects. General clinical considerations include LILRB4 presence on non-malignant myeloid cells, which, when selectively targeted, may disrupt normal immune functions. Additionally, in one case outside the context of a clinical trial (NCT05038800, terminated), a patient with refractory AML experienced acute myocarditis after receiving a single dose of mAb MK-0482, which may have been linked to unknown effects of LILRB4 inhibition on immune regulation [143, 147]. These observations underline the need for careful monitoring and thorough safety evaluation of LILRB4-targeted therapies in future studies.

### Lymphocyte antigen 9 (LY9)

#### Biological functions

Lymphocyte antigen 9 (LY9), otherwise known as SLAMF3 or CD229, is a member of the SLAM family of receptors. These receptors play critical roles in regulating adaptive and innate immune responses, including leukocyte differentiation, activation, and cytokine secretion [148]. LY9 is a cell surface glycoprotein, with four Ig-like domains. Like other SLAMF receptors, LY9 is activated by homophilic interactions, meaning it binds to itself as a ligand. This self-binding interaction transmits a strong positive signal to T cells by recruiting SLAM-associated adaptor proteins [149, 150].

Under normal physiological conditions, LY9 is expressed in T and B lymphocytes, monocytes, plasma cells, and other immune cells [151]. However, in the context of MM, LY9 is overexpressed on malignant plasma cells across various disease stages, including monoclonal gammopathy of undetermined significance (MGUS), smoldering MM, MM, extramedullary MM, and plasma cell leukemia [152, 153]. The effects of LY9 in MM are mediated through the MAPK/ERK signaling pathway, which involves adaptor proteins (e.g., SHP2 and GRB2) promoting cell proliferation and survival [149].

In addition to its membrane-bound form, soluble LY9 has been detected at high levels in aggressive forms of MM. Elevated soluble LY9 correlates with shorter progression-free survival, suggesting that it protects malignant cells from spontaneous apoptosis and contributes to disease progression [152, 154, 155].

#### Therapeutic potential and challenges

Given its elevated expression in MM cells and involvement in disease progression, LY9 has been examined as a therapeutic target in preclinical studies. Anti-LY9 CAR T cells exert robust cytotoxicity against MM cells in vitro. Although LY9 is also expressed on normal T cells, functional T cells are typically LY9-negative, which reduces the risk of CAR T-cell fratricide and improves the feasibility of this approach [156]. Additionally, fine-tuning anti-LY9 CAR T cells, such as introducing a single amino acid substitution in the CAR-binding domain, has further minimized the risk of targeting healthy lymphocytes expressing low levels of LY9. This modification has been combined with the overexpression of c-Jun, which enhances CAR T cell activity and durability [157]. These findings suggest that LY9 CAR T cells could be a promising therapeutic option for MM patients, particularly those with advanced or refractory disease.

However, potential challenges remain. One issue is antigen escape, a phenomenon in which LY9 expression on MM cells may decrease or disappear during treatment, as observed for other MM markers like BCMA [158]. The use of LY9-targeted therapies in combination with other established MM treatments could enhance efficacy and reduce the risk of antigen escape, providing a more robust therapeutic strategy. Another concern is that anti-LY9 CAR T cells may upregulate their own surface expression of LY9 over time, potentially impairing their function or leading to unintended fratricide. Moreover, LY9 and other SLAMF members have been reported to mitigate their phagocytosis by macrophages under certain circumstances, enabling immune evasion [159]. These limitations highlight the need for careful monitoring and optimization of LY9-targeted therapies.

Clinical trials specifically targeting LY9 in MM have not yet been initiated, highlighting a significant gap in clinical research that warrants further exploration. While preclinical data are promising, additional studies are needed to evaluate the safety and efficacy of LY9-targeted therapies in vivo and to determine the most effective therapeutic strategies for clinical application.

### Semaphorin 4 A (SEMA4A)

#### Biological functions

Semaphorin 4 A (SEMA4A) is a class 4 semaphorin family member, which typically exists as a membrane-bound receptor [160]. SEMA4A is a single-pass type I membrane protein, characterized by structural domains, including an immunoglobulin-like C2-type domain, a plexin–semaphorin–integrin domain, and a Sema domain, which facilitate its diverse biological roles [161]. Proteolytic cleavage of SEMA4A generates soluble forms that regulate immune responses and influence various pathophysiological conditions [162].

SEMA4A interacts with a range of receptors, including class B plexins, Plexin D1, T-cell immunoglobulin domain and mucin domain-2 (TIMD-2), neuropilin-1 (NRP1), and immunoglobulin like transcript 4 (ILT4), which mediate different signaling outcomes, mainly related to immune cell activation, differentiation, and function [161]. For example, SEMA4A expressed by DCs co-stimulates Th lymphocyte proliferation. In humans, this drives Th2 responses via ILT4 receptor binding, whereas in mice, it promotes Th1 responses through TIMD-2 binding, facilitating immune activation [163]. Within the TME, tumor-derived SEMA4A can activate cytotoxic T cells, contributing to anti-tumor immune responses [164]. In addition to its roles in normal immune function, SEMA4A has been implicated in various autoimmune-related disorders and malignancies [161, 165].

#### Therapeutic potential and challenges

SEMA4A has been identified as a novel immunotherapeutic target in MM, based on findings from unbiased proteomics studies. These studies reveal that SEMA4A expression levels in MM cells are higher compared to those of other common MM targets, such as BCMA or SLAMF7 [11, 166]. Preclinical studies of anti-SEMA4A CAR T-cell therapy have demonstrated the effective elimination of SEMA4A-expressing MM cells, highlighting its therapeutic potential [166]. Moreover, Anderson et al. demonstrated that SEMA4A is essential for MM cell survival [11], suggesting that it is less likely to be lost through genetic deletions, a common mechanism of acquiring therapy resistance in MM.

Although SEMA4A expression can decrease following short-term treatment with bortezomib [12], its levels remain sufficient in many cases to serve as a viable therapeutic target. Furthermore, the low levels of soluble SEMA4A in MM patients are unlikely to interfere with antibody-based therapies, as soluble SEMA4A is not expected to significantly compete with a membrane-bound form for antibody binding [11]. The potential for SEMA4A internalization upon ligand binding also makes it a suitable candidate for ADCs, which rely on rapid internalization to deliver cytotoxic payloads effectively [11]. Since MM patients often have a compromised immune system due to the disease and its treatments, SEMA4A-targeting ADCs could offer an advantage, as their action does not depend on an intact immune response.

Despite promising preclinical data, the development of SEMA4A-targeted therapies faces several challenges. Antigen loss or downregulation could lead to a relapse or reduced efficacy, as observed with other targets like BCMA. Moreover, SEMA4A expression on normal immune cells, such as activated T cells, DCs, monocytes, and granulocytes, could result in off-target effects, limiting the therapeutic window [10, 165]. Beyond MM, SEMA4A plays roles in immune evasion and TME modulation in other cancers, highlighting its broad potential as an oncological target [164, 165]. Further research is needed to clarify its functions and therapeutic implications.

Overall, SEMA4A is a compelling immunotherapy target for MM, due to its high expression and functional relevance in MM cells. Continued exploration of CAR T cells, ADCs, and other modalities targeting SEMA4A may yield novel treatment strategies, both as monotherapies and combined with existing MM therapies. Optimizing these approaches will be crucial to maximizing efficacy while minimizing off-target effects.

### Solute carrier family 3 member 2 (SLC3A2)

#### Biological functions

Solute carrier family 3 member 2 (SLC3A2), also known as CD98hc, is a transmembrane glycoprotein and a key member of the heterodimeric amino acid transporters (HAT) family. SLC3A2 is a single-pass heavy chain linked via a disulfide bond to one of six possible multipass light chains, such as the large neutral amino acid transporter 1 (LAT1) [167]. The heavy and light chains create a transmembrane protein complex that regulates intracellular calcium levels and amino acid transport. This complex plays a crucial role in “outside-in” integrin signaling, through interactions involving its intracellular and transmembrane domains in many cell types [168, 169].

The CD98hc heavy chain promotes integrin-mediated signaling by binding to various β1 and β3 integrins, thereby enhancing integrin-dependent processes, such as cell spreading, migration, growth, and survival, ultimately protecting against apoptosis [168, 170, 171]. SLC3A2 has also been associated with the mTOR signaling pathway [172] and the unfolded protein response [173], suggesting its involvement in regulating cellular growth and stress responses. Blocking SLC3A2 disrupts these adhesive signals, representing a promising strategy for interfering with tumor cell interactions within the microenvironment [134, 168, 171].

SLC3A2 is overexpressed in several cancers, including prostate cancer, bladder cancer, papillary thyroid carcinoma, colorectal carcinoma, and gastric cancer, where its upregulation is linked to worse patient outcomes [174]. Overexpression of SLC3A2 promotes anchorage-independent cell growth, tumorigenesis, and activation of integrin-regulated signaling pathways, making it a critical player in cancer progression [170].

#### Therapeutic potential and challenges

In MM, SLC3A2 is highly expressed on malignant cells and can be specifically targeted by mAbs. For example, the mAb R8H283 binds to SLC3A2 heterodimers on MM cells but does not bind normal hematopoietic or non-hematopoietic cells [175]. R8H283 specifically recognizes SLC3A2 in complex with its light chains, without reacting to SLC3A2 monomers, which are less abundant on normal cells. On MM cells, the unique glycoforms of CD98 heterodimers facilitate selective targeting, positioning R8H283 as a promising candidate for antibody-based therapies in MM [175]. Even though possible targets in the form of CD98hc heterodimers are present in normal leukocytes, R8H283 does not bind to those [175]. In addition, the overexpression of LAT1, which forms a complex with SLC3A2, is associated with upregulated proliferation of malignant cells and poorer prognosis in MM patients. RNAi-mediated depletion of CD98hc has been shown to decrease cell proliferation [176, 177].

The transporter function of SLC3A2 is dispensable for MM cell survival and proliferation, whereas its integrin-related signaling appears to be crucial. This signaling is also essential for the rapid proliferation of B and T cells, leading to clonal expansion and plasma cell differentiation [178, 179]. Consequently, therapeutic targeting of SLC3A2 poses a potential clinical challenge due to its critical role in normal immune and epithelial cell functions. Although an in vivo murine study showed that SLC3A2 inhibition did not adversely affect the already developed hematopoietic system [180], its safety profile in humans remains unclear. This concern is particularly relevant for T and NK cell functions, as SLC3A2 facilitates amino acid transport and supports mTOR signaling [181].

Nevertheless, SLC3A2 appears to be a viable target for MM immunotherapy due to its high expression on MM cells and its role in promoting survival and proliferation. Future development of immunotherapeutic modalities, such as mAbs or CAR T-cell therapies, could leverage this target to disrupt MM cell interactions with the TME, while minimizing off-target effects on healthy tissues.

### TNF receptor superfamily member 13 C (TNFRSF13C)

#### Biological functions

TNFRSF member 13 C (TNFRSF13C), also called B-cell activating factor receptor (BAFFR), is a member of the TNFRSF family involved in B-cell survival and differentiation. Unlike typical TNFRSF members, which contain several extracellular CRDs for ligand binding and receptor complex assembly, TNFRSF13C has only a partial extracellular CRD. This truncated structure still enables ligand binding and self-assembly but structurally distinguishes TNFRSF13C from other family members [182].

B-cell activating factor (BAFF, also referred to as TNFSF13B or CD257), the primary ligand of TNFRSF13C, activates critical signaling pathways, including NF-κB, PI3K, and ERK, which are essential for B-cell survival, protein synthesis, and metabolic fitness. TNFRSF13C primarily triggers the canonical NF-κB1 and non-canonical NF-κB2 signaling pathways [182, 183]. Although BAFF also interacts with other receptors, such as transmembrane activator and CAML interactor (TACI) and BCMA, its role in signaling through TNFRSF13C is distinct and critical [184].

Following BAFF binding, TNFRSF13C undergoes proteolytic cleavage by metalloproteases ADAM10 and ADAM17, reducing its cell surface expression [182, 184, 185]. Interestingly, the cleaved extracellular portion of TNFRSF13C likely remains bound to BAFF, reducing the possibility of functioning as a decoy receptor. Once BAFF dissociates from soluble TNFRSF13C, the receptor loses its ligand-binding capability, terminating its function [182].

TNFRSF13C expression is restricted to B cells, with levels increasing as B cells mature. It plays a critical role in B-cell survival and maturation by interacting with BAFF [182]. Dysregulation of TNFRSF13C can disrupt B-cell development, leading to conditions such as B lymphopenia, hypogammaglobulinemia, and impaired humoral immunity. Polymorphisms in the TNFRSF13C gene are associated with immunodeficiencies, autoimmune disorders, and B-cell lymphomas [184].

#### Therapeutic potential and challenges

Therapeutic strategies targeting TNFRSF13C aim to downregulate aberrant NF-κB signaling or neutralize its ligand, BAFF [186]. VAY-736, an anti-TNFRSF13C mAb, was developed to block TNFRSF13C-BAFF interactions and has shown promising antitumor effects in chronic lymphocytic leukemia models, particularly when combined with ibrutinib, a Bruton tyrosine kinase inhibitor [186, 187]. Targeting TNFRSF13C with anti-TNFRSF13C antibodies has shown potential in MM treatment by inducing apoptosis in the KM-3 MM cell line [183].

TNFRSF13C has been detected in MM patient samples, with its expression observed in two-thirds of freshly isolated samples in one study [188]. However, the same study reported a lack of TRNFSR13C protein expression in five tested MM cell lines, raising questions about its expression profile in MM. The authors suggested that the absence of TNFRSF13C in cultured cell lines might result from in vitro conditions or indicate in vivo loss of expression. Despite these inconsistencies, the limited available data suggest that targeting TNFRSF13C may still be a viable approach in MM therapy.

Given the critical role of TNFRSF13C in B-cell development and homeostasis, developing TNFRSF13C-targeting therapies will require careful consideration to avoid adverse effects on normal B-cell function. Nonetheless, its unique expression pattern and role in B-cell signaling make TNFRSF13C a promising candidate for further investigation as a therapeutic target in MM and other B-cell malignancies.

## Promising candidates and broader target evaluation

Despite significant advancements in immunotherapeutic strategies, the treatment of MM continues to face substantial challenges. FDA-approved immunotherapies targeting BCMA, GPRC5D, CD38, and SLAMF7 have demonstrated significant efficacy, yet several limitations persist. For instance, antigen escape, where MM cells downregulate or mutate targeted antigens, reduces treatment durability and response rates [4, 158, 189–192]. Additionally, soluble forms of surface antigens and shedding contribute to immune evasion and may hinder drug efficacy, while off-tumor toxicity raises safety concerns. The heterogeneous nature of MM and its complex TME further complicates therapeutic targeting, as variability in antigen expression across disease stages undermines the consistency of surface antigen-based approaches [193]. These barriers underscore the need for innovative strategies to overcome current limitations and optimize MM immunotherapies.

**Fig. 2:**
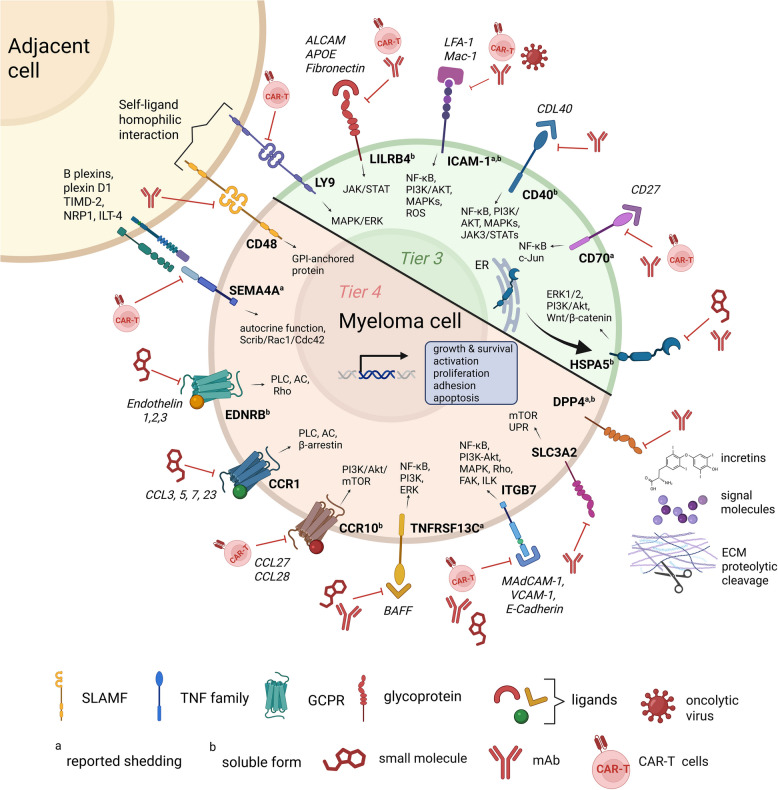
Potential immunotherapy candidates for MM. This schematic illustration highlights novel immunotargets expressed on the surface of MM cells, their ligands, associated signaling pathways, and potential functional impacts. Key targets, such as LILRB4, SEMA4A, ITGB7 and others, are depicted within critical signaling pathways, including NF-κB, PI3K/AKT, and JAK/STAT, which drive the growth, survival, and proliferation of malignant plasma cells. Clinically relevant modalities, such as CAR T cells and small molecule inhibitors that modulate these immunotargets are emphasized, showcasing the complexity and therapeutic potential of these receptors in MM treatment. (Illustration created with BioRender.com)

The advent of surface proteomics and integrative omics technologies has significantly advanced the identification and validation of novel MM immunotargets [11–14]. These techniques facilitate a comprehensive analysis of surface antigens, revealing targets with high specificity and stability that traditional methods may have overlooked [194]. Emerging targets, such as LILRB4, SEMA4A, ITGB7, CD70, and CCR1, demonstrate unique biological relevance and therapeutic promise (Fig. 2, Table 2). However, their clinical translation hinges on robust preclinical validation to ensure tumor specificity, stability of expression, and minimal off-tumor effects [195, 196].

**Table 2:** Key ligands, pathways, and treatment modalities summarized for the explored MM immunotargets (updated on February 5, 2025)

Immunotarget	Ligands	Biological functions	Signaling pathway	Therapeutic modalities against immunotarget	Soluble form or shedding
CD40	CD40L [17, 18]	B-cell and dendritic cell activation, germinal center formation, and class-switched antibody generation [16]	NF-κB, PI3K/AKT, MAPKs, and JAK3/STATs [17, 18]	mAbs SGN-40 (dacetuzumab) [21], Lucatumumab [23], XmAbCD40 [24]	Soluble forms [19, 20]
CD70	CD27 [29]	Lymphocyte maturation and proliferation [31, 34]	NF-κB, c-Jun [32]	mAbs cusatuzumab (ARGX-110) (NCT01813539), IMM40H [32], SGN-70 [40], CAR-T [30, 32]	Shedding of CD70 has not been reported. On the other hand, shedding of its receptor CD27 has been described [32]
HSPA5	n/a	Protein folding, assembly, and degradation [42]	ERK1/2, PI3K/AKT, or Wnt/β-catenin [48]	Small molecule inhibitor HA15 [42], and mAb PAT-SM6 [52]	A soluble form [51]
ICAM-1	LFA-1, Mac-1 [56]	Leukocyte recruitment in inflammation. Immune synapse formation during T-cell activation [59]	NF-κB, PI3K/AKT, MAPKs, ROS, and Src family kinases [197]	Oncolytic virus (NCT05698888), mAb (NCT01025206, NCT02756728), and CAR-T [198]	Shedding and soluble form [199]
CCR1	Mainly CCL3, CCL5, CCL7, and CCL23 (summarized in [64])	Bone and MM TME, immune cell migration [65, 66]	PLC, AC, and β-arrestin-related pathways [66]	Small molecule antagonists CCCCX354-C (NCT01242917) and BX-471 (NCT00185341)	n/a
CCR10	CCL27 and CCL28	T-cell trafficking and responses to viral infection [80]	PI3K/AKT/mTOR signaling pathway [79]	CAR-T [12]	n/a
CD48	Ligand for CD2 and SLAMF4 [88]	Co-stimulatory and regulatory functions in immune cells, such as T, B, and NK cells [87, 89]	GPI-anchored protein, receptor reverse signaling not described [87–89]	Blocking mAb [94], and ADC SGN-CD48A [95]	Shedding reported to be increased in pathological conditions [87]
DPP4	Varied bioactive peptides (incretins, hormones, etc.) [101]	T-cell activation and carcinogenesis [104]	Proteolytic enzyme [100]	mAbs IF7, 14D10 and huCD26mAb [104]	Circulating soluble form, shedding in cancer [106, 107]
EDNRB	Endothelin-1, −2, −3 [108]	Vascular homeostasis [108]	PLC, AC, and Rho pathways [108, 109]	Small molecule inhibitor BQ-788 (NCT02442466), and dual EDNRA/EDNRB antagonists macitentan and bosentan [117, 118]	n/a
ITGB7	MAdCAM-1, VCAM-1 and E-Cadherin [124]	Trafficking and homing of immune cells, facilitating lymphocyte migration [124]	NF-κB, PI3K/AKT, MAPK, Rho, FAK, ILK [200]	Small molecule inhibitor TRK-170 (NCT01345799), and mAb vedolizumab and CAR-T OPC-415 (NCT04649073)	n/a
LILRB4	ALCAM, APOE, and fibronectin [135, 142, 201]	Tumor cell infiltration and T-cell activity suppression [139]	JAK/STAT signaling pathway [139]	mAbs IO-202 (NCT0437243, NCT05309187), NGM831 (NCT05215574), MK-0482 (NCT03918278), ADC [138], CAR-T [143], STAR-T (NCT05518357), and bispecific T-cell engager [14]	A soluble form [137]
LY9	Homophilic interactions, self-ligand [149]	Leukocyte differentiation, activation, and cytokine secretion [154]	MAPK/ERK signaling pathway [149]	CAR-T [156]	A soluble form [155]
SEMA4A	Interacts with B plexins, Plexin D1, TIMD-2, NRP1, and ILT-4 [161]	Neuronal and immunological regulation, including dendritic cell function and T-cell activation, differentiation, and function [163, 164]	Not well understood and cell type-dependent, ligand has autocrine functions as Plexin B1 receptor: inhibition of DC migration via Scrib/Rac1/Cdc42 pathway [161, 165]	CAR-T [166], and ADC [11]	Functional soluble forms exist but shedding is reported as minimal in MM [11]
SLC3A2	n/a	Integrin-dependent cell spreading, migration, growth, and survival by promoting adhesive signals and protecting cells from apoptosis [168, 170, 171]	mTOR signaling and unfolded protein response [173]	mAb R8H28 [175]	n/a
TNFRSF13C	BAFF	B-cell survival, protein synthesis, and metabolic fitness [186]	NF-κB, PI3K, and ERK [183]	mAb VAY-736 [187]	Shedding [184]

n/a data not available

Among the emerging targets evaluated in this review, several stand out for their potential to complement or enhance existing MM therapies (Fig. 1, Supplementary Table S1, Fig. 2, Table 2). LILRB4, SEMA4A, ITGB7, CD70, and CCR1 are particularly promising based on their selective expression, roles in MM pathogenesis, and accessibility for therapeutic targeting (Supplementary Table S1, Table 3). For instance, SEMA4A is implicated in TME modulation and immune suppression, making it a compelling candidate for combination therapies to overcome immune evasion [11, 161, 164]. Similarly, the role of CD70 in promoting lymphocyte proliferation and its selective overexpression in high-risk MM subtypes, such as extramedullary disease, positions it as a strong candidate for both CAR-T and antibody-based therapies, with additional potential for combination strategies aimed at reducing immune evasion through CD27 signaling modulation [32, 34]. LILRB4, with its T-cell suppressive effects, also highlights the potential for immune activation and tumor control [139, 143]. Similarly, CCR1 and ITGB7 contribute to critical tumor-stroma interactions that drive MM cell migration, adhesion, and drug resistance, emphasizing their therapeutic relevance [124, 125]. However, further preclinical and early clinical studies are essential to validate their functional relevance and optimize therapeutic strategies.

**Table 3:** Summary of key emerging targets in MM immunotherapy

Target	Rationale for Inclusion and Potential Impact	Known Limitations	Safety Profile
CD70	Homogeneous and specific expression in MM cells, plays a significant role in immune evasion and plasma cell proliferation	Shedding of its receptor (CD27) may reduce efficacy; off-tumor expression on activated immune cells poses a risk	No shedding reported for CD70 itself, though CD27 shedding has been observed; strong preclinical and clinical safety demonstrated in antibody-based therapies
CCR1	Overexpression in MM correlates with poor prognosis and enhanced dissemination; disrupting CCR1 signaling could reduce metastases and tumor progression	Exhibits high ligand promiscuity that may increase risk of resistance mechanisms. Expression is noted in some inflammatory conditions	No soluble form detected; preclinical safety data suggest selective targeting may avoid significant immune suppression or adverse effects
ITGB7	Central role in CAM-DR and immune evasion, promoting tumor adhesion to bone marrow stroma; disrupting ITGB7 interactions could help overcome resistance mechanisms	Limited clinical evidence in MM; most data derive from solid tumors or inflammatory conditions. Off-tumor expression on lymphocytes poses potential risks	Favorable safety profile reported from anti-ITGB7 agents in inflammatory diseases; no significant adverse effects noted in MM-specific studies
LILRB4	Implicated in T-cell suppression, immune evasion, and MM cell proliferation; promising preclinical data with mAbs and CAR T	Potential off-target effects due to expression in myeloid cells	Soluble form detected; acute myocarditis reported in one case with an anti-LILRB4 mAb in a non-MM setting; other preclinical and clinical studies reported a safety profile, supporting selective targeting in MM
SEMA4A	High expression in MM cells with a critical role in TME modulation, and immune suppression; strong candidate for ADC and CAR T-cell therapies	Risk of antigen downregulation and off-tumor expression in activated immune cells	Low levels of soluble form in MM patients suggest minimal therapeutic interference; preclinical studies indicate low off-target toxicity

While these top-tier candidates in Table 3 demonstrate strong potential, other targets described in this review, such as CCR10 and CD48, merit further exploration despite not being highlighted in this summary table. Both CCR10 and CD48 were consistently identified in all four studies reviewed, underscoring their potential relevance as immunotargets even with lower individual rankings. This consistency highlights the therapeutic opportunities these targets present, particularly when a cross-study validation is considered. LY9, with its homogenous and specific expression across MM stages, also offers strong rationale for further preclinical evaluation. HSPA5 stands out for its involvement in protein homeostasis and association with treatment-resistant quiescent MM cells. At the same time, the dual role of ENDRB in tumor biology and immune regulation suggests promise for combination strategies. These examples illustrate that even outside the top-ranked targets, there is significant scope for further research and therapeutic development.

It is important to acknowledge that scoring systems and methodologies varied among the studies evaluated, making direct comparisons challenging. However, the diversity of biological functions and expression patterns observed among these novel targets strengthens the rationale for continued exploration and validation. For example, the selective expression of CCR10 in MM cells compared to healthy peripheral blood cells and the role of CD48 in immune synapse formation suggest they could serve as valuable complementary targets. These features collectively offer diverse opportunities for therapeutic innovation in MM and underline the need for strategic prioritization in preclinical and clinical development efforts.

## Conclusions and future directions

The emerging landscape of MM immunotargets identified through recent surfaceomics and integrative omics studies offers new opportunities to advance immunotherapy. This review highlights targets, such as LILRB4, SEMA4A, ITGB7, CD70, and CCR1. These candidates demonstrate the potential to address key challenges in MM treatment, including antigen escape, treatment resistance, and off-tumor toxicity.

To translate these emerging targets into clinical practice, future efforts must prioritize rigorous preclinical validation, rational combination strategies, and the development of predictive biomarkers to support patient stratification in clinical trials. It will be essential to evaluate the stability and tumor specificity of target antigen expression across disease stages and treatment contexts.

Moreover, assessing target expression homogeneity, safety profiles, and potential resistance mechanisms should be an integral part of the preclinical validation pipeline to mitigate off-tumor effects and treatment failure. In parallel, combination strategies, such as integrating CAR T cells with checkpoint inhibitors or bsAbs, may enable the simultaneous targeting of distinct surface markers or mechanisms of action, thereby promoting more comprehensive tumor eradication, even in heterogeneous MM populations. Future studies should investigate optimal combinations and evaluate their clinical benefits. Additionally, challenges related to resistance mechanisms, including antigen loss, soluble antigen interference, and immune evasion, must be addressed to achieve durable therapeutic responses. Accordingly, emerging immunotargets should be assessed for their potential to overcome these barriers, either as stand-alone therapy or in combination with other modalities.

Altogether, continued efforts in target validation, clinical optimization, and translational research are essential to fully realize the potential of these promising candidates and improve long-term outcomes for MM patients.

## Supplementary Information

Supplementary Material 1.

## Data Availability

No datasets were generated or analysed during the current study.

## Abbreviations

α2M: Alpha-2-macroglobulin
AC: Adenylyl cyclase
ADC: Antibody-drug conjugate
AML: Acute myeloid leukemia
APC: Antigen-presenting cell
BAFF: B-cell activating factor
BAFFR: B-cell activating factor receptor
BCMA: B-cell maturation antigen
bsAb: Bispecific antibody
CAM-DR: Cell-adhesion-mediated drug resistance
CAR: Chimeric antigen receptor
CCL3: C-C Motif Chemokine Ligand 3
CCR1: CC Motif Chemokine Receptor 1
CD: Cluster of differentiation
CMML: Chronic myelomonocytic leukemia
CRD: Cysteine-rich domain
CX3CL1: C-X3-C motif chemokine ligand 1
CX3CR1: C-X3-C motif chemokine receptor 1
DC: Dendritic cell
DPP4: Dipeptidyl peptidase 4
EDNRB: Endothelin receptor type B
EGFR: Epidermal growth factor receptor
EMT: Epithelial-mesenchymal transition
ER: Endoplasmic reticulum
FDA: U.S. Food and Drug Administration
GPCR: G protein-coupled receptor
GPRC5D: Like G protein-coupled receptor class C group 5 member D
GRP78: Glucose-regulated protein 78
HAT: Heterodimeric amino acid transporters
HIF-2α: Hypoxia-inducible factor 2α
HSPA5: Heat shock protein family A (Hsp70) member 5
ICAM-1: Intercellular adhesion molecule 1
ILT3: Immunoglobulin-like transcript 3
ITGB7: Integrin subunit beta 7
ITIMs: Immunoreceptor tyrosine-based inhibitory motifs
LAT1: Large neutral amino acid transporter 1
LILRB4: Leukocyte immunoglobulin-like receptor B4
LY9: Lymphocyte antigen 9
mAb: Monoclonal antibody
MAdCAM-1: Mucosal vascular addressing cell adhesion molecule 1
MGUS: Monoclonal gammopathy of undetermined significance
MM: Multiple myeloma
n/a: Not applicable
NK: Natural killer
NRP1: Neuropilin-1
PAR-4: Prostate apoptosis response 4
PD-1: Programmed death-1
PLC: Phospholipase C
R/R: Relapsed/refractory
SEMA4A: Semaphorin 4A
SLAMF7: Signaling lymphocytic activation molecule family member 7
SLC3A2: Solute carrier family 3 member 2
TACI: Transmembrane activator and CAML interactor
TCR: T-cell receptor
TIMD-2: Immunoglobulin domain and mucin domain-2
TME: Tumor microenvironment
TNF: Tumor necrosis factor
TNFRSF: TNF receptor superfamily
V-CAM: Vascular cell adhesion molecule
